# Development and validation of methods to predict ground- and helicopter-based emergency medical service travel times

**DOI:** 10.1186/s13049-026-01722-1

**Published:** 2026-11-02

**Authors:** Douglas Spangler, Julia Sheffield, Johan Alm, David Smekal, Jyrki Tenhunen, Annelie Barrueta Tenhunen

**Affiliations:** 1 Anesthesiology and Intensive Care Medicine, Department of Surgical Sciences, Uppsala University, Uppsala, Sweden; 2 Department of Ambulance- and Emergency Care, Uppsala University Hospital, Uppsala, Sweden; 3 Department of Intensive Care Medicine and HEMS, Uppsala University Hospital, Uppsala, Sweden

## Abstract

**Background:**

Helicopter-based Emergency Medical Services (EMS) are a costly and finite resource and should only be deployed when they provide substantial benefits to patients. One such benefit is a more rapid response to incidents and transport to hospital compared to Ground-based EMS. This study evaluates methods for estimating travel times for the purpose of optimizing the selection of helicopter vs. ground EMS.

**Methods:**

Ground EMS travel times were estimated using an open-source routing tool, the estimates of which were adjusted using linear regression and gradient boosting models based on historical travel times. Helicopter travel times were similarly estimated based on great circle distances adjusted by historical data. Models were evaluated using several measures of predictive accuracy to enable comparison with other studies.

**Results:**

In validation data collected after finalization of the methods, the best performing prediction models for Ground EMS travel times explained a substantial portion of variation in drive times to location and to hospital (R2 0.79 and 0.70 respectively), and less for helicopter flight times (R2 0.46 and 0.38 respectively). Mean average percentage error was 18-20% for all investigated estimation techniques and time intervals.

**Conclusion:**

Integrating tools based on the open-source code provided with this study has the potential to improve the ability of dispatchers to estimate relative travel times and assess the potential time benefit of directing the most appropriate resources to a given patient.

**Supplementary Information:**

The online version contains supplementary material available at https://doi.org/10.1186/s13049-026-01722-1.

## Background

Helicopter Emergency Medical Services (HEMS) are a costly and finite resource that should be deployed when they offer a clear advantage over ground EMS (GEMS). One potential advantage of HEMS is shorter travel times, which have been associated with improved patient outcomes across multiple acute conditions [1–8]. However, estimating the potential travel-time advantage of HEMS over GEMS for a given incident is not straightforward, as travel times depend on multiple factors including distance, road quality, weather, traffic, ambulance availability, and station placement [5, 9].

Several studies have compared GEMS and HEMS travel times, generally showing that HEMS are faster over longer distances, and in many cases provide shorter total to-hospital times [4, 10–12]. Some studies have attempted to define geographical cutoffs for when HEMS may be faster than GEMS [11, 13], but these cutoffs tend to be context-specific and difficult to generalize to other EMS systems [14].

Current HEMS dispatch criteria are based primarily on estimated clinical need [15–17], and physician-staffed HEMS has demonstrated a severity-adjusted mortality benefit compared to GEMS [18–20]. However, even in the absence of a need for more advanced prehospital interventions, shorter to-incident and to-hospital times may benefit patients in the highest-priority dispatch category. Some systems already incorporate travel time predictions into dispatch decision-making [21].

Prediction models for EMS planning have been explored previously [22, 23]. One study using a machine learning algorithm to predict GEMS to-incident times in Stockholm found low overall correlation with actual times, though correlation was better among high-priority dispatches [9]. A generalizable, easily implementable method for estimating and comparing GEMS and HEMS travel times remains lacking.

We hypothesized that it would be possible to develop a method for estimating GEMS and HEMS travel times, and that such a method could identify local geographical thresholds at which HEMS has a travel-time advantage over GEMS. We further aimed to provide open-source code that could be readily implemented in other prehospital systems to inform dispatch decision-making.

### Aim

To develop and validate methods for estimating ground and helicopter-based EMS travel times, with the goal of identifying potential time savings associated with HEMS.

## Methods

### Study design

A single-center model development study with validation in a dataset collected subsequent to the finalization of the models.

### Study setting

This study was conducted in Uppsala, Sweden, a mixed urban-rural region spanning 12 173 km and containing 410 572 residents in 2025. The region is serviced by a single regional, hospital-based EMS agency fielding 13 daytime ambulances across 4 ambulance stations and an additional 4 consistently manned stand-by posts. HEMS responses are provided by a single hospital-based intensive care HEMS service based near the main hospital to which the majority of critical patients are transported. HEMS is typically dispatched based on indication by the nurse staffed Emergency Medical Dispatch center responsible for prehospital care in the region. Ground EMS are staffed by at least one nurse and either a second nurse or an emergency medical technician, while HEMS units are staffed clinically by a medical doctor and a nurse. The analyses performed in this study generally assume simultaneous dispatch of HEMS and GEMS resources.

### Data collection

Data for model development was collected from the dispatching system in Region Uppsala for the years of 2022 to 2024, supplemented by data collected from HEMS flight logs. Ground ambulance missions were filtered to include only missions with a priority 1 (lights and sirens) response. In analyzing to-hospital times, only those relating to priority 1 patient transports were included. GEMS missions missing a documented ambulance starting point, and HEMS missions in which the helicopter was not at base at the time of dispatch were excluded, as well as missions with an at-scene time of less than 1 min or more than 3 h, as these were generally the result of documentation errors by ambulance crews. Hourly data on temperature, wind, precipitation, snow depth, and sight distance was downloaded from The Swedish Meteorological and Hydrological Institute [24]. Subsequent to finalization of the travel time prediction models, similar data was collected for 2025 to serve as a validation dataset. Data collection for this study was approved by the Swedish Ethics Review Authority (dnr 2024-08307-01).

### Travel time prediction

Baseline travel time and distance estimates for ground ambulance missions were generated using the Open Service Routing Machine (OSRM) [25]. Using this tool, the distance and estimated drive time from the starting point to the incident location, as well as from the incident to the receiving hospital was estimated. Baseline travel distances for HEMS missions were estimated based on Great Circle distances between the starting location and incident, and between the incident and hospital using the *sf* R package [26]. Estimates were generated both for the 2022–2024 training period, and the 2025 validation period under the assumption that ORSM would be available in a real-world application.

These baseline times and distances were then used as predictors in linear regression models estimating travel times during the training period as a function of the baseline distance and time (including an interaction effect to capture potential differences in estimated and actual driving speed across road types), mission priority (higher acuity 1 A or lower acuity 1B), time of day (using a sin and cosine transformation to capture variation across the day and including an interaction with the distance and time terms), the starting location of the call, the type of call (Medical, Trauma, or traffic accident), and the distance of the incident to the nearest road included in the OSRM road network data. Weather variables were similarly included as linear predictors in the HEMS models, and interactions with drive time estimates were included in the GEMS models. In addition to the linear regression models, estimates based on non-parametric gradient boosting were generated using the XGBoost package, with all interactions and non-linear effects handled natively by the machine learning algorithm. One of each type of model for each combination of HEMS / GEMS and to-incident / to-hospital times, for a total of 8 evaluated models. The exact specification of the models is available in Supplementary materials 1.

### Statistical analysis

Predicted travel times estimated as described above were compared with actual travel times in the 2025 validation dataset to evaluate model accuracy. To enable comparison with other research, model performance was evaluated in terms of multiple performance metrics including absolute deviation, Mean Absolute Percentage Error (MAPE), R^2^ value (i.e. proportion of variation in travel times explained by the predictions), and Root Mean Square Error (RMSE). Scatterplots were used to visualize predicted vs. true travel times to evaluate calibration. Travel times were visualized geographically by plotting travel times on a map using thin plate spline regression to interpolate travel times between observed values using the *fields* R package [27]. Descriptive statistics were generated using appropriate measures of central tendency and 1000 bootstrap replicates were used to generate 95% confidence intervals. All analysis was performed using R v 4.4.2 [28] and all code and models used to perform this analysis is included as Supplementary material 1, and is available in the online repository associated with this study [29].

## Results

A total of 24 756 incidents were identified meeting the study inclusion criteria, of which 527 were responded to by HEMS. The characteristics of these incidents are provided in Table 1 below. It may be seen that HEMS missions tend to be higher priority, more often related to trauma and traffic accidents, and are further away from both the nearest ambulance station and from the main receiving hospital.

**Table 1:** Descriptive statistics

	Ground EMS		Helicopter EMS	
	Training set	Validation set	Training set	Validation set
Number of included incidents	17,604	6546	304	183
Priority 1 A*	7.6 (7.2-8)	7.6 (7-8.2)	42.4 (36.8–48.4)	38.8 (31.7–45.9)
Medical incidents*	86.3 (85.7–86.8)	86.3 (85.4–87.1)	58.9 (53.3–64.5)	65.6 (58.5–72.1)
Trauma incidents*	11 (10.5–11.5)	11.2 (10.5–12)	22.4 (17.8–27.3)	23 (16.9–29)
Traffic Accident incidents**	2.8 (2.5-3)	2.5 (2.1–2.9)	18.8 (14.5–23)	11.5 (7.1–16.4)
Estimated driving distance to incident (km)**	10 (9.9–10.2)	10 (9.7–10.2)	19.6 (18.4–20.8)	21 (19.6–22.7)
Estimated driving distance to hospital (km)**	27.7 (27.4–28.1)	26.5 (25.9–27.2)	55.3 (53-57.6)	51.5 (48.2–55.1)
Time to location (minutes)**	10.8 (10.7–10.9)	10.7 (10.5–10.8)	23.6 (22.8–24.5)	22.4 (21.2–23.6)
Time to hospital (minutes)**	53.2 (52.6–53.8)	52.8 (51.8–53.8)	64 (61.9–65.9)	63.2 (60.4–65.9)

* Percentages

** Mean values

Mean / Percentage values are presented with bootstrapped 95% Confidence Intervals

### Prediction models

Gradient Boosting (GB) and Linear regression Models (LM) were fit to estimate travel times to the incident and the total to-hospital time for GEMS and HEMS respectively. The characteristics of each of these models are presented in Table 2 below, and the full model specification for each may be found in supplementary materials 1. The GEMS models had a generally high degree of explained variance, while there was more unexplained variability in the HEMS models. The models were able to predict travel times to the incident with an average absolute error of ca. 1.7 min for GEMS and ca. 4.5 min for HEMS, with greater errors estimating total time to hospital. As a percentage of the total time however, all models performed similarly, with mean average percentage errors of around 18–20%. In terms of R^2^, the models tended to capture more of the variation in the data regarding GEMS (70%-79%), than for HEMS missions (35%-46%).

**Table 2:** Model performance characteristics

Type	Destination	Method	Mean true time	Absolute deviation	MAPE	R^2^	RMSE
GEMS	To hospital	GB	52.8	8.56	17.9	0.704	12.13
	To hospital	LM	52.8	8.76	18.8	0.703	12.18
	To location	GB	10.7	1.58	17.4	0.793	2.83
	To location	LM	10.7	1.68	19.2	0.774	2.95
HEMS	To hospital	GB	63.2	10.57	18.4	0.355	13.33
	To hospital	LM	63.2	10.54	18.3	0.384	12.67
	To location	GB	22.4	4.55	20.6	0.438	6.3
	To location	LM	22.4	4.49	21	0.463	6.12

GEMS: Ground Emergency Medical Services, HEMS: Helicopter Emergency Medical Services, GB: Gradient Boosting, LM: Linear Model, MAPE: Mean Absolute Percentage Error, R^2^: Coefficient of Determination, RMSE: Root Mean Squared Error

**Fig. 1:**
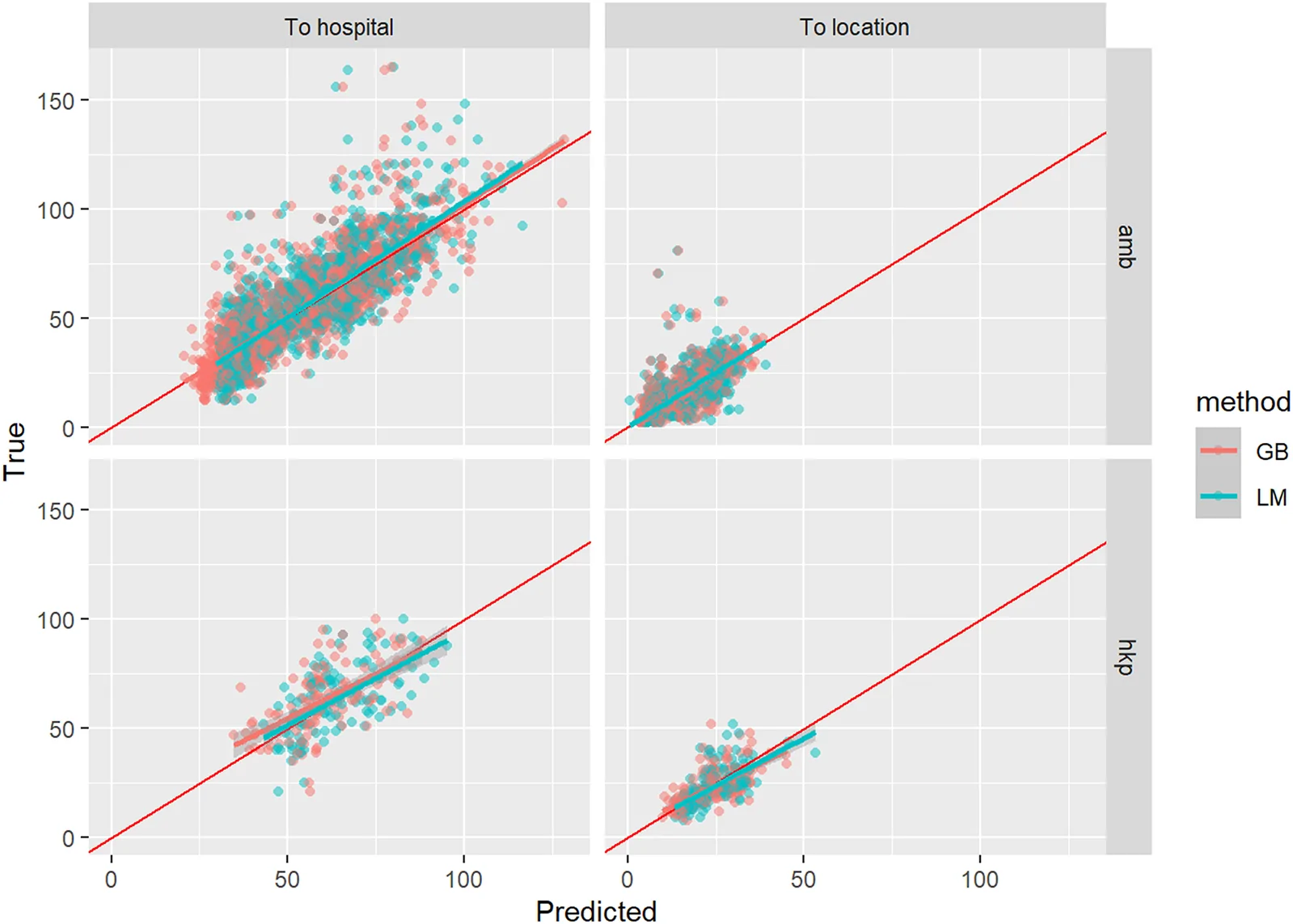
Predicted vs. true travel times. Each dot represents a travel time prediction and true value, with the red diagonal line representing the line of perfect fit. Lines of best fit are drawn for each model to illustrate calibration. GB: Gradient Boosting, LM: Linear Model

For each of these models, plots of predicted vs. true historical travel times were plotted to reveal patterns in model predictions as shown in Fig. 1. As may be seen in the figure, the predictions generally fit true values well, though some extreme low and high travel time values exist which are not well characterized by the models, though these may also be due to mis-documented times.

**Fig. 2:**
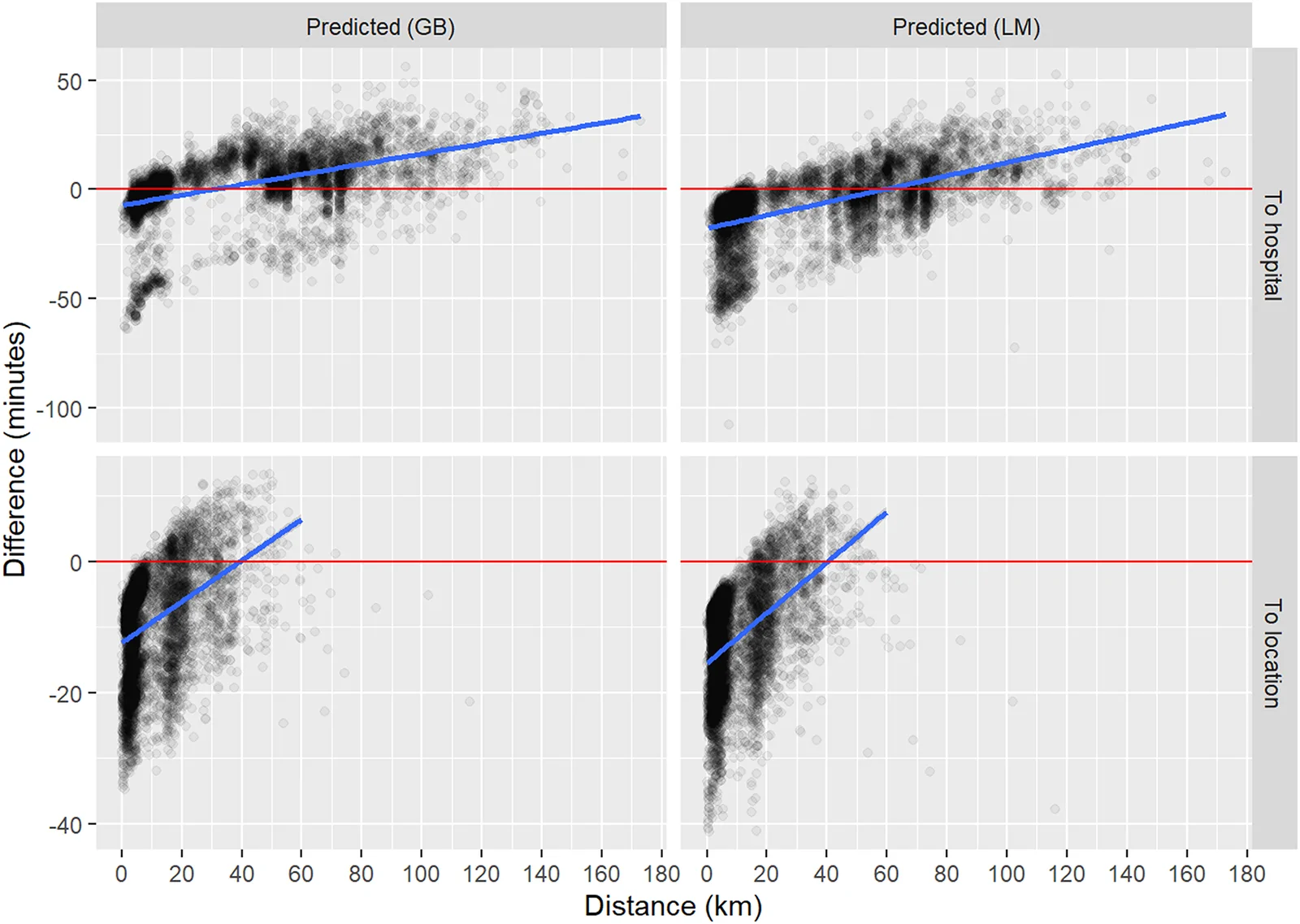
HEMS vs. GEMS time gain vs. road distance travelled GB: Gradient Boosting, LM: Linear Model

Figure 2 illustrates the relationship between the road distance to the incident/total distance travelled to the hospital. While there is substantial variability stemming from other variables accounted for in the models, it may be seen that benefits in terms of time reductions become apparent around 50 km. Note also the non-linear behavior of the GB models at short to-hospital distances, stemming partly from the lack of observations for HEMS responses at these short distances.

**Fig. 3:**
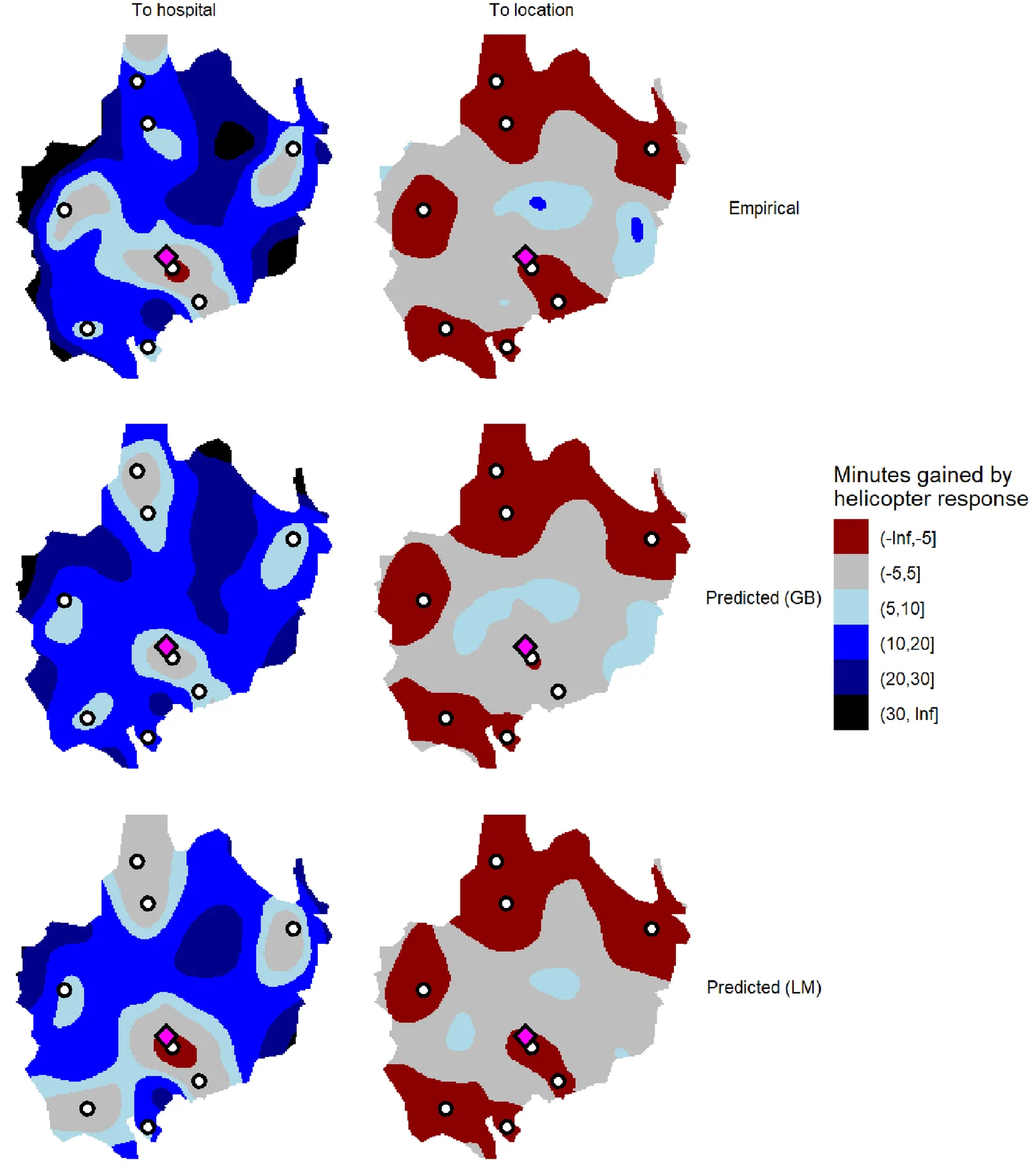
Predicted and empirical travel time differences between ground and helicopter EMS. Travel time estimates interpolated using thin plate splines, with white dots representing the location of ambulance stations and the magenta diamond representing the location of the helicopter base. The main receiving hospital is co-located at the black dot near the helicopter base. Predictions of the Gradient Boosting (GB) and Linear Regression model (LM) are shown separately

The model predictions may finally be used to compare the difference in travel times geographically, estimating travel times using the models for each ground and helicopter mission. The resulting travel time differences based on the prediction models may be compared to differences between travel times directly interpolated from historical data, as shown in Fig. 3:

It may be seen that while the empirically generated and predicted travel time difference estimates generally coincide well, there are some discrepancies, e.g. in the far east and north of the region. Overall, there are few parts of the region where a HEMS response generally reduces to-incident times, although substantial areas in the northwest and northeast where HEMS has the potential to substantially reduce transport times to the hospital.

## Discussion

The prediction models we present provide useful method for estimating​, in particular GEMS travel times. The models generally explained more of the variability in GEMS times than HEMS times, suggesting a greater degree of variability stemming from factors not captured in the model. In prospective application, both regression and gradient boosting-based approaches had similar levels of accuracy. As applied to the studied region, there were few geographic areas where HEMS has an overall potential to reduce to-incident times, but in many outlying areas, reductions in total to-hospital times of over 10 min could be expected, with substantial expected benefits in terms of time savings beginning at road travel distances of around 40 km to incidents, and at 50 to 60 km of total road travel distances to hospital. Per most evaluation metrics, the models had a greater ability to predict GEMS times than HEMS times, which may be due to the more detailed routing data available in estimating GEMS times, and to more substantial variability in both HEMS preparation time and on-scene time stemming from e.g. the number and intensity of interventions performed by HEMS crews [30].

We compared two approaches to estimating travel times, using a traditional linear regression approach and a non-parametric machine learning algorithm. Both approaches had similar predictive accuracy, with a slight edge for the non-parametric GB approach per most metrics. While regression modelling requires some thoughtfulness in considering which interaction and non-linear effects to include (which is not necessary using the machine learning approach), it is also more consistent in training and validation data, and may extrapolate better to new situations (e.g. missions with very short transport distances as suggested by the clustering seen in Fig. 2). While the weather-related parameters added a small amount of predictive value (constituting a larger proportion of the models total explanatory power for HEMS times), model performance is primarily based on geographical and temporal data, and these auxiliary predictors may not be worth the additional complexity necessary to incorporate them in a real-time predictive tool. A full accounting of the importance of each included variable may be found in Supplementary materials 1.

The performance of the models reported here can be difficult to compare with other approaches, given the strong influence of the study setting on model performance. The R^2^ of 0.79/0.77(for GB / LM models respectively) for the GEMS to-incident time model compares favorably with that developed by Hill et al. which achieved an R^2^ of 0.37, although those results also included lower-priority calls [9]. The MAPE of 17.8/18.8% for patient to-hospital times found here similarly outperformed the 45% MAPE reported by Abid et al. [31]. Similarly, the MAPE for to-incident times of 17.2/19.2% identified here compares favorably to the 25% identified by Fleschman et al. [32]. Meanwhile, the model proposed by Westgate et al. achieved a substantially lower RMSE (72 s vs. 198 as found here), though these results were identified in the highly restrictive context of urban Toronto with response distances of < 5 km [22]. We identified no previous literature attempting to predict HEMS to-incident times, and thus lack benchmarks regarding the performance of our approach. Stassen et al. however investigated the distance at which a HEMS response is warranted in the context of HEMS requested by on-scene GEMS, which contrasts with the setting of this study in which GEMS and HEMS are typically dispatched simultaneously. Stassen et al. identified a driving distance of 119 km at which HEMS results in reduced to-hospital times, which corresponds roughly to our findings of a travel-time threshold of 50 km, given that our results include GEMS drive times both to and from the incident [33].

We encourage researchers to make use of the source code provided with this study and further develop the methods we report. Further efforts may for instance include the use of real-time traffic data which would could improve the GEMS model fit.

### Limitations

The use of HEMS in primary incident response is motivated not only by shortened travel times, but also by the enhanced competencies provided by on-scene medical doctors typically found on HEMS units. As such, findings relating to low potential for time savings in incident responses should not be construed as an argument against the impact of on-scene HEMS care overall. Our model is intended to estimate the potential travel-time advantage of HEMS over GEMS, rather than to determine whether HEMS is clinically indicated for a given patient.

It should also be noted that there may be differences in the duration of the interval from the documented time of arrival to the hospital until the patient arrives to definitive care. In our setting, GEMS units deliver the patient directly to the Emergency Department, while HEMS units must transport the patient from the helicopter landing pad, which can add up to 10 min to the total time it takes for a patient to be handed over to definitive care. Similar limitations exist regarding to-incident times, as the time from arrival at the location to patient contact is not documented for either HEMS or GEMS resources. While we lack data to model these intervals, in a practical application they could be represented by adding flat time penalties to the estimated helicopter or ambulance travel time.

Several methodological limitations should be noted. Data were restricted to high priority missions transported to the primary regional hospital, limiting generalizability to other transport modes and destinations. It may also be noted that especially at the extreme high end of travel times, travel times tended to be under-estimated, although the possibility of these extreme values being due to documentation errors cannot be excluded. It was noted that the volume of HEMS responses was substantially higher in the validation dataset, though this was verified as reflecting a true increase in the number of primary HEMS responses. We also excluded HEMS and GEMS missions not originating from a documented point of departure, as GPS data regarding their position at time of dispatch was not available. While we provide the models reported here for demonstration purposes, any real implementation of our methods should be done after re-training the models based on data collected in the local context, and accounting for status and position at time of dispatch.

## Conclusion

The open-source methods presented here provide estimates of GEMS and HEMS travel times competitive with state-of-the-art methods. If implemented in clinical practice along with robust tools for establishing the need for the higher medical competency provided by HEMS teams, these estimation techniques could serve as part of an effective decision support tool for dispatchers to aid in determining the potential time benefit of directing HEMS resources to a given patient.

## Supplementary Information

Below is the link to the electronic supplementary material.

Supplementary Material 1

## Data Availability

Access to individual level data is available to researchers with appropriate Swedish ethics approval upon contact with ambulanssjukvard@akademiska.se. All code necessary to replicate the results is available in an online repository at https://doi.org/10.17605/OSF.IO/NUGV4.

## Abbreviations

EMS: Emergency Medical Services
HEMS: Helicopter Emergency Medical Services
GEMS: Ground Emergency Medical Services
OSRM: Open Service Routing Machine
MAPE: Mean Average Percentage Error
RMSE: Root Mean Square Error
GB: Gradient Boosting
LM: Linear regression Model

## Abbreviations

EMS: Emergency Medical Services
HEMS: Helicopter Emergency Medical Services
GEMS: Ground Emergency Medical Services
OSRM: Open Service Routing Machine
MAPE: Mean Average Percentage Error
RMSE: Root Mean Square Error
GB: Gradient Boosting
LM: Linear regression Model
