# Supplementary material for: Development and validation of methods to predict ground- and helicopter-based emergency medical service travel times
Source: Scand J Trauma Resusc Emerg Med. 2026 Nov 2;34:170. doi: 10.1186/s13049-026-01722-1 (PMC13737524; doi:10.1186/s13049-026-01722-1)
Supplement: Supplementary file 1 [file 13049_2026_1722_MOESM1_ESM.html]

Supplement 1 - Analysis notebook for ‘Development and validation of methods to predict ground- and helicopter-based emergency medical service travel times’


# Supplement 1 - Analysis notebook for ‘Development and validation of methods to predict ground- and helicopter-based emergency medical service travel times’

#### Douglas Spangler

# Setup session

```
library(tidyverse)
library(sf)
library(sp)
library(gstat)
library(knitr)
library(osrm)
library(xgboost)
library(fields)
library(terra)

knitr::opts_chunk$set(echo = TRUE)
options(scipen = 999,
        digits = 3)

projcrs <- "+proj=longlat +datum=WGS84 +no_defs +ellps=WGS84 +towgs84=0,0,0"

ci_bootstrap <- function(x, 
                         fun,
                         conf_level = 0.95, 
                         n_bootstrap = 1000,
                         na.rm = T,
                         print = F,
                         pct = F,
                         r=1) {
  
  # Generate ordinary bootstrap estimates of CI for descriptive stats
  
  est = fun(x,na.rm = na.rm)
  
  boots <- replicate(n_bootstrap, {
    sample_x <- sample(x, 
                       length(x), 
                       replace = TRUE)
    fun(sample_x,na.rm = na.rm)
  })
  lower_bound <- quantile(boots, 
                          (1 - conf_level) / 2)
  upper_bound <- quantile(boots, 
                          1 - (1 - conf_level) / 2)
  if(pct){
    est = est*100
    lower_bound = lower_bound*100
    upper_bound = upper_bound*100
  }
  
  if(print){
    return(paste0(round(est,r)," (",round(lower_bound,r),"-",round(upper_bound,r),")"))
  }else{
    return(c(est,lower_bound, upper_bound))
  }
  
}

data_dictionary = c("id" = "Unique case identifier",
                    "latitude" = "GPS latitude (WGS 84) of incident location", 
                    "longitude" = "GPS longitude (WGS 84) of incident location", 
                    "AssignedDT" = "Timestamp when unit was assigned", 
                    "PrioIn" = "Priority to hospital", 
                    "PrioOut" = "Priority to incident", 
                    "StartPlace" = "Starting location (station)",
                    "CloseDestination" = "Destination hospital", 
                    "true_time_loc" = "Actual time to location", 
                    "true_time_hosp" = "Actual time to hospital", 
                    "type" = "'amb' for GEMS or 'hkp' for HEMS", 
                    "CallType" = "Type of call", 
                    "StartLatitude" = "GPS latitude (WGS 84) of EMS at start of call", 
                    "StartLongitude" = "GPS longitude (WGS 84) of EMS at start of call", 
                    "EndLongitude" = "GPS latitude (WGS 84) of destination", 
                    "EndLatitude" = "GPS longitude (WGS 84) of destination", 
                    "precipitation" = "Precipation (mm/hr) at time of unit assignment", 
                    "temperature" = "Temperature (celcius) at time of unit assignment", 
                    "windspeed" = "Windspeed (m/s) at time of unit assignment", 
                    "winddirection" = "Wind direction (degrees) at time of unit assignment", 
                    "snowdepth" = "Snow depth (m) at time of unit assignment, measured daily and interpolated", 
                    "visibility" = "Visibility (m) at time of unit assignment")
```

# Data collection

Data for all ambulance incidents with a priority 1 response to and
from an incident with a documented starting location, as well as all
primary helicopter responses. Data was collected regarding the position
of the incident, call characteristics (time, type of call, and
priority), and combined with hourly weather data.

```
#Lots of data parsing excluded here, but available in a seperate data generation script
load("combined_df.rda")

# Variable description
kable(data.frame("Description" = data_dictionary))
```

|  | Description |
| --- | --- |
| id | Unique case identifier |
| latitude | GPS latitude (WGS 84) of incident location |
| longitude | GPS longitude (WGS 84) of incident location |
| AssignedDT | Timestamp when unit was assigned |
| PrioIn | Priority to hospital |
| PrioOut | Priority to incident |
| StartPlace | Starting location (station) |
| CloseDestination | Destination hospital |
| true\_time\_loc | Actual time to location |
| true\_time\_hosp | Actual time to hospital |
| type | ‘amb’ for GEMS or ‘hkp’ for HEMS |
| CallType | Type of call |
| StartLatitude | GPS latitude (WGS 84) of EMS at start of call |
| StartLongitude | GPS longitude (WGS 84) of EMS at start of call |
| EndLongitude | GPS latitude (WGS 84) of destination |
| EndLatitude | GPS longitude (WGS 84) of destination |
| precipitation | Precipation (mm/hr) at time of unit assignment |
| temperature | Temperature (celcius) at time of unit assignment |
| windspeed | Windspeed (m/s) at time of unit assignment |
| winddirection | Wind direction (degrees) at time of unit assignment |
| snowdepth | Snow depth (m) at time of unit assignment, measured daily and interpolated |
| visibility | Visibility (m) at time of unit assignment |

# Analysis

Ambulance response data was augmented using the Open Source Routing
machine based on Open Street Maps data to determine the distance from
the ambulance station to the incident, and the distance from the
incident to the receiving hospital. An estimate of the duration
(i.e. drive time) for a typical car following speed limits was also
extracted from the service, as well as an estimate of the distance to
the nearest road.

## Generate car routing data

Note: Change eval= FALSE to TRUE when re-training models (Takes a
long time!)

```
# Use demo server (Ok to use for small batches, but for larger datasets, will need to set up your own server)
# options(osrm.server = "https://router.project-osrm.org/")

# Use local instance of OSRM
options(osrm.server = "http://localhost:5000/")

options(osrm.profile = 'car') 

## Get routes ------

combined_df_routes <- combined_df 

combined_df_routes$distance_nearest <- NA
combined_df_routes$drive_loc_duration <- NA
combined_df_routes$drive_loc_distance <- NA

combined_df_routes$drive_hosp_duration <- NA
combined_df_routes$drive_hosp_distance <- NA

for(i in 1:nrow(combined_df_routes)) {
  d <- combined_df_routes[i,]
  drive_loc <- osrmRoute(src = select(d,StartLongitude,StartLatitude),
                         dst = select(d,longitude,latitude))
  
  drive_hosp <- osrmRoute(src = select(d,longitude,latitude),
                          dst = select(d,EndLongitude,EndLatitude))
  
  distance <- osrmNearest(select(d,longitude,latitude))
  
  combined_df_routes$distance_nearest[i] <- distance$distance
  combined_df_routes$drive_loc_duration[i] <- drive_loc$duration
  combined_df_routes$drive_loc_distance[i] <- drive_loc$distance
  
  combined_df_routes$drive_hosp_duration[i] <- drive_hosp$duration
  combined_df_routes$drive_hosp_distance[i] <- drive_hosp$distance
  
  combined_df_routes$drive_tot_duration[i] <- drive_loc$duration + drive_hosp$duration
  combined_df_routes$drive_tot_distance[i] <- drive_loc$distance + drive_hosp$distance
  if(i%%100==0) print(i)
  
}

save(combined_df_routes,file = "combined_df_routes.rda")
```

```
load("combined_df_routes.rda")

combined_df_routes <- combined_df_routes %>%
  st_as_sf(x = .,
           coords = c("longitude", "latitude"),
           remove = F,
           crs = projcrs)%>%
  filter(true_time_loc < 1.5*60,
         true_time_hosp < 3*60 | is.na(true_time_hosp),
         true_time_loc > 2,
         true_time_hosp > 10 | is.na(true_time_hosp)) %>%
  mutate(set = ifelse(year(AssignedDT)<=2024,"train","test"),
         drive_tot_duration = drive_loc_duration+drive_hosp_duration,
         drive_tot_distance = drive_loc_distance+drive_hosp_distance,
         time_numeric = as.numeric(hms::as_hms(AssignedDT)),
         time_theta = 2*pi*time_numeric / (24*3600),
         WeekDay = weekdays(AssignedDT),
         distance_loc = as.numeric(st_distance(.,st_as_sf(st_drop_geometry(.), 
                                                          coords = c("StartLongitude", 
                                                                     "StartLatitude"), 
                                                          crs = projcrs),
                                               by_element = T))/1000,
         distance_hkp = as.numeric(st_distance(.,st_as_sf(st_drop_geometry(.), 
                                                          coords = c("HkpLongitude", 
                                                                     "HkpLatitude"), 
                                                          crs = projcrs),
                                               by_element = T))/1000,
         distance_hosp = as.numeric(st_distance(.,st_as_sf(st_drop_geometry(.), 
                                                           coords = c("EndLongitude", 
                                                                      "EndLatitude"), 
                                                           crs = projcrs),
                                                by_element = T))/1000,
         distance_tot = distance_hkp + distance_hosp)

combined_df_routes <- filter(combined_df_routes,
                             excl_base == 0 | is.na(excl_base))

train_df_routes <- combined_df_routes %>%
  filter(set == "train")

val_df_routes <- combined_df_routes %>%
  filter(set == "test")


train_xgb_data <- st_drop_geometry(train_df_routes) %>%
  transmute(latitude,
            longitude,
            PrioOut = as.numeric(factor(PrioOut)),
            CallType = as.numeric(factor(CallType)),
            StartPlace = as.numeric(factor(StartPlace)),
            WeekDay = as.numeric(as.factor(WeekDay)),
            time_theta,distance_hkp,distance_hosp,distance_tot,
            precipitation,temperature,windspeed,snowdepth,visibility,
            distance_nearest,drive_loc_duration,drive_loc_distance,
            drive_hosp_duration,drive_hosp_distance,
            drive_tot_duration,drive_tot_distance) %>%
  as.matrix()


val_xgb_data <- st_drop_geometry(val_df_routes) %>%
  transmute(latitude,
            longitude,
            PrioOut = as.numeric(factor(PrioOut)),
            CallType = as.numeric(factor(CallType)),
            StartPlace = as.numeric(factor(StartPlace)),
            WeekDay = as.numeric(as.factor(WeekDay)),
            time_theta,distance_hkp,distance_hosp,distance_tot,
            precipitation,temperature,windspeed,snowdepth,visibility,
            distance_nearest,drive_loc_duration,drive_loc_distance,
            drive_hosp_duration,drive_hosp_distance,
            drive_tot_duration,drive_tot_distance) %>%
  as.matrix()
```

## Descriptive data

```
combined_df_routes %>%
  group_by(type,set) %>%
  st_drop_geometry() %>%
  summarise(n = as.character(n()),
            pct_prio_1a = ci_bootstrap(PrioOut == "1A",mean,pct=T,print=T),
            pct_type_medical = ci_bootstrap(CallType == "Medical",mean,pct=T,print=T),
            pct_type_trauma = ci_bootstrap(CallType == "Trauma",mean,pct=T,print=T),
            pct_type_traffacc = ci_bootstrap(CallType == "TrafficAccident",mean,pct=T,print=T),
            mean_distance_loc = ci_bootstrap(drive_loc_distance,mean,print=T),
            mean_distance_hosp = ci_bootstrap(drive_hosp_distance,mean,print=T),
            mean_time_loc = ci_bootstrap(true_time_loc,mean,print=T),
            mean_time_hosp = ci_bootstrap(true_time_hosp,mean,print=T)) %>%
  pivot_longer(-c(type,set)) %>%
  pivot_wider(names_from = "type") %>%
  kable()
```

```
## `summarise()` has regrouped the output.
## ℹ Summaries were computed grouped by type and set.
## ℹ Output is grouped by type.
## ℹ Use `summarise(.groups = "drop_last")` to silence this message.
## ℹ Use `summarise(.by = c(type, set))` for per-operation grouping
##   (`?dplyr::dplyr_by`) instead.
```

| set | name | amb | hkp |
| --- | --- | --- | --- |
| test | n | 6546 | 183 |
| test | pct\_prio\_1a | 7.6 (7-8.2) | 38.8 (31.7-45.9) |
| test | pct\_type\_medical | 86.3 (85.4-87.1) | 65.6 (58.5-72.1) |
| test | pct\_type\_trauma | 11.2 (10.5-12) | 23 (16.9-29) |
| test | pct\_type\_traffacc | 2.5 (2.1-2.9) | 11.5 (7.1-16.4) |
| test | mean\_distance\_loc | 10 (9.7-10.2) | 21 (19.6-22.7) |
| test | mean\_distance\_hosp | 26.5 (25.9-27.2) | 51.5 (48.2-55.1) |
| test | mean\_time\_loc | 10.7 (10.5-10.8) | 22.4 (21.2-23.6) |
| test | mean\_time\_hosp | 52.8 (51.8-53.8) | 63.2 (60.4-65.9) |
| train | n | 17604 | 304 |
| train | pct\_prio\_1a | 7.6 (7.2-8) | 42.4 (36.8-48.4) |
| train | pct\_type\_medical | 86.3 (85.7-86.8) | 58.9 (53.3-64.5) |
| train | pct\_type\_trauma | 11 (10.5-11.5) | 22.4 (17.8-27.3) |
| train | pct\_type\_traffacc | 2.8 (2.5-3) | 18.8 (14.5-23) |
| train | mean\_distance\_loc | 10 (9.9-10.2) | 19.6 (18.4-20.8) |
| train | mean\_distance\_hosp | 27.7 (27.4-28.1) | 55.3 (53-57.6) |
| train | mean\_time\_loc | 10.8 (10.7-10.9) | 23.6 (22.8-24.5) |
| train | mean\_time\_hosp | 53.2 (52.6-53.8) | 64 (61.9-65.9) |

## Model estimation

The OSRM-augmented data was then used to predict response and
transport times using linear regression models. All models estimate the
response time to the location or hospital (total time from initial call
to arrival at hospital) based on the distance to the incident (road
distance for ground ambulances and straight-line distance for
helicopters) or total distance to incident plus distance to hospital,
respectively. All models include time of day modeled using sin/cosine
transformations. Ambulance drive time models also include the priority
of the call, the starting location of the ambulance, and the distance of
the incident from the nearest road included in the OSRM routing topology
as covariates. For ground ambulances, both the distance and estimated
duration (i.e. drive time) based on OSRM are included, as well an an
interaction term between them to represent possible variation in the
degree to which ambulances are able to exceed posted speed limits.
Weather data was found to primarily impact the base travel time for HEMS
models, while these terms were found to interact strongly with distances
for ground ambulances (i.e. affect driving speed).

Note: change eval= FALSE to TRUE when re-training models

```
drive_model_loc = glm(true_time_loc ~  (drive_loc_duration * drive_loc_distance) *
                        (sin(time_theta) * cos(time_theta)) + 
                        drive_loc_duration:precipitation+ 
                        drive_loc_duration:snowdepth +
                        drive_loc_duration:visibility +
                        drive_loc_duration:windspeed +
                        drive_loc_duration:temperature +
                        PrioOut +
                        StartPlace + 
                        CallType +
                        WeekDay +
                        distance_nearest,
                      family = gaussian(link = "log"),
                      data = filter(st_drop_geometry(train_df_routes),
                                    type == "amb"),
                      model = FALSE)

fly_model_loc = glm(true_time_loc ~ distance_hkp * 
                      (sin(time_theta) * cos(time_theta)) + 
                      precipitation + 
                      snowdepth +
                      visibility +
                      windspeed +
                      temperature +
                      distance_nearest,
                    family = gaussian(link = "log"),
                    data = filter(st_drop_geometry(train_df_routes),
                                  type == "hkp"),
                    model = FALSE)

drive_model_hosp = glm(true_time_hosp ~ (drive_tot_duration * drive_tot_distance) +
                         (sin(time_theta) + cos(time_theta))  + 
                         drive_loc_duration:precipitation + 
                         drive_loc_duration:snowdepth +
                         drive_loc_duration:visibility +
                         drive_loc_duration:windspeed +
                         drive_loc_duration:temperature + 
                         PrioOut + 
                         StartPlace + 
                         CallType +
                         WeekDay +
                         distance_nearest,
                       family = gaussian(link = "log"),
                       data = filter(st_drop_geometry(train_df_routes),
                                     type == "amb"),
                       model = FALSE)

fly_model_hosp = glm(true_time_hosp ~ distance_tot * 
                       (sin(time_theta) * cos(time_theta)) + 
                       precipitation + 
                       snowdepth +
                       visibility +
                       windspeed +
                       temperature +
                       PrioOut +
                       CallType,
                     family = gaussian(link = "log"),
                     data = filter(st_drop_geometry(train_df_routes),
                                   type == "hkp"),
                     model = FALSE)


drive_gbcvmodel_loc = xgb.cv(label = train_df_routes$true_time_loc[train_df_routes$type == "amb"],
                             nfold=5,
                             nrounds = 500,
                             early_stopping_rounds = 10,
                             prediction = T,
                             data = train_xgb_data[train_df_routes$type == "amb",])

drive_gbmodel_loc = xgboost(label = train_df_routes$true_time_loc[train_df_routes$type == "amb"],
                            nrounds = drive_gbcvmodel_loc$best_iteration,
                            data = train_xgb_data[train_df_routes$type == "amb",])

drive_gbcvmodel_hosp = xgb.cv(label = train_df_routes$true_time_hosp[train_df_routes$type == "amb" &
                                                                       !is.na(train_df_routes$true_time_hosp)],
                              nfold=5,
                              nrounds = 500,
                              early_stopping_rounds = 10,
                              prediction = T,
                              data = train_xgb_data[train_df_routes$type == "amb" &
                                                      !is.na(train_df_routes$true_time_hosp),])

drive_gbmodel_hosp = xgboost(label = train_df_routes$true_time_hosp[train_df_routes$type == "amb" &train_df_routes$type == "amb" & !is.na(train_df_routes$true_time_hosp)],
                             nrounds = drive_gbcvmodel_hosp$best_iteration,
                             data = train_xgb_data[train_df_routes$type == "amb" &
                                                     !is.na(train_df_routes$true_time_hosp),])


fly_gbcvmodel_loc = xgb.cv(label = train_df_routes$true_time_loc[train_df_routes$type == "hkp"],
                           nfold=5,
                           nrounds = 500,
                           early_stopping_rounds = 10,
                           prediction = T,
                           data = train_xgb_data[train_df_routes$type == "hkp",])

fly_gbmodel_loc = xgboost(label = train_df_routes$true_time_loc[train_df_routes$type == "hkp"],
                          nrounds = fly_gbcvmodel_loc$best_iteration,
                          data = train_xgb_data[train_df_routes$type == "hkp",])

fly_gbcvmodel_hosp = xgb.cv(label = train_df_routes$true_time_hosp[train_df_routes$type == "hkp" &
                                                                     !is.na(train_df_routes$true_time_hosp)],
                            nfold=5,
                            nrounds = 500,
                            early_stopping_rounds = 10,
                            prediction = T,
                            data = train_xgb_data[train_df_routes$type == "hkp" &
                                                    !is.na(train_df_routes$true_time_hosp),])

fly_gbmodel_hosp = xgboost(label = train_df_routes$true_time_hosp[train_df_routes$type == "hkp" &train_df_routes$type == "hkp" & !is.na(train_df_routes$true_time_hosp)],
                           nrounds = fly_gbcvmodel_hosp$best_iteration,
                           data = train_xgb_data[train_df_routes$type == "hkp" &
                                                   !is.na(train_df_routes$true_time_hosp),])


models <- list("lm_drive_loc" = drive_model_loc,
               "lm_drive_hosp" = drive_model_hosp,
               "lm_fly_loc" = fly_model_loc,
               "lm_fly_hosp" = fly_model_hosp,
               
               "gb_drive_loc" = drive_gbmodel_loc,
               "gb_drive_hosp" = drive_gbmodel_hosp,
               "gb_fly_loc" = fly_gbmodel_loc,
               "gb_fly_hosp" = fly_gbmodel_hosp)

save(models,file="models.rda")
```

```
load("models.rda")

pred_df <- val_df_routes %>%
  mutate(pred_drive_loc = predict(models$lm_drive_loc, newdata = .,type = "response"),
         predgb_drive_loc = predict(models$gb_drive_loc,newdata = val_xgb_data),
         pred_drive_hosp = predict(models$lm_drive_hosp, newdata = .,type = "response"),
         predgb_drive_hosp = predict(models$gb_drive_hosp,newdata = val_xgb_data),
         pred_fly_loc = predict(models$lm_fly_loc, newdata = .,type = "response"),
         predgb_fly_loc = predict(models$gb_fly_loc,newdata = val_xgb_data),
         pred_fly_hosp = predict(models$lm_fly_hosp, newdata = .,type = "response"),
         predgb_fly_hosp = predict(models$gb_fly_hosp,newdata = val_xgb_data),
         pred_time_loc = ifelse(type == "amb",pred_drive_loc,pred_fly_loc),
         pred_time_hosp = ifelse(type == "amb",pred_drive_hosp,pred_fly_hosp),
         predgb_time_loc = ifelse(type == "amb",predgb_drive_loc,predgb_fly_loc),
         predgb_time_hosp = ifelse(type == "amb",predgb_drive_hosp,predgb_fly_hosp),
         pred_diff_loc = pred_drive_loc - pred_fly_loc,
         pred_diff_hosp = pred_drive_hosp - pred_fly_hosp,
         predgb_diff_loc = predgb_drive_loc - predgb_fly_loc,
         predgb_diff_hosp = predgb_drive_hosp - predgb_fly_hosp,
         id=row_number())

# summary(drive_model_loc)
# summary(fly_model_loc)
# summary(drive_model_hosp)
# summary(fly_model_hosp)
```

## Model performance

```
rsq <- function (x, y) cor(x, y,use="pairwise.complete.obs") ^ 2
rmse <- function(x, y) sqrt(mean((x - y)^2,na.rm=T))
absdev <- function(x, y) mean(abs(x - y),na.rm=T)
mape <- function(x, y) mean(abs((x - y) / x)*100,na.rm=T)

truevpred <- select(pred_df,
                    id,
                    StartPlace,
                    type,
                    "True_time_To location_LM" = true_time_loc,
                    "True_time_To hospital_LM" = true_time_hosp,
                    "Predicted_time_To location_LM" = pred_time_loc,
                    "Predicted_time_To hospital_LM" = pred_time_hosp,
                    "True_time_To location_GB" = true_time_loc,
                    "True_time_To hospital_GB" = true_time_hosp,
                    "Predicted_time_To location_GB" = predgb_time_loc,
                    "Predicted_time_To hospital_GB" = predgb_time_hosp) %>%
  st_drop_geometry() %>%
  pivot_longer(-c(type,id,StartPlace)) %>%
  separate(name,sep = "_",into = c("est","time","destination","method")) %>%
  pivot_wider(names_from = est,
              values_from = value,
              id_cols=c(type,destination,time,id,StartPlace,method))

truevpred %>%
  group_by(type,destination,method) %>%
  summarise(mean_true = mean(True,na.rm=T),
            absdev = absdev(True,Predicted),
            mape = mape(True,Predicted),
            rsq = rsq(True,Predicted),
            rmse = rmse(True,Predicted)) %>%
  kable()
```

```
## `summarise()` has regrouped the output.
## ℹ Summaries were computed grouped by type, destination, and method.
## ℹ Output is grouped by type and destination.
## ℹ Use `summarise(.groups = "drop_last")` to silence this message.
## ℹ Use `summarise(.by = c(type, destination, method))` for per-operation
##   grouping (`?dplyr::dplyr_by`) instead.
```

| type | destination | method | mean\_true | absdev | mape | rsq | rmse |
| --- | --- | --- | --- | --- | --- | --- | --- |
| amb | To hospital | GB | 52.8 | 8.56 | 17.9 | 0.704 | 12.13 |
| amb | To hospital | LM | 52.8 | 8.76 | 18.8 | 0.703 | 12.18 |
| amb | To location | GB | 10.7 | 1.58 | 17.4 | 0.793 | 2.83 |
| amb | To location | LM | 10.7 | 1.68 | 19.2 | 0.774 | 2.95 |
| hkp | To hospital | GB | 63.2 | 10.57 | 18.4 | 0.355 | 13.33 |
| hkp | To hospital | LM | 63.2 | 10.54 | 18.3 | 0.384 | 12.67 |
| hkp | To location | GB | 22.4 | 4.55 | 20.6 | 0.438 | 6.30 |
| hkp | To location | LM | 22.4 | 4.49 | 21.0 | 0.463 | 6.12 |

## Model predicitve plots

Models are generally well calibrated vs true times

Note generally longer preparation and on-scene times for helicopter
responses (blue).

```
truevpred %>%
  ggplot(aes(x=Predicted,y=True,color = method)) +
  geom_point(alpha = 0.5,shape = 16) +
  facet_grid(type~destination) +
  geom_abline(color = "red") +
  #scale_color_brewer(palette = "Set3") +
  geom_smooth(method = "lm") +
  expand_limits(xmin = 0,
                ymin=0)
```

```
## `geom_smooth()` using formula = 'y ~ x'
```

```
## Warning: Removed 9618 rows containing non-finite outside the scale range
## (`stat_smooth()`).
```

```
## Warning: Removed 9618 rows containing missing values or values outside the scale range
## (`geom_point()`).
```

## Times vs distance

Plots illustrating the distance at which helicopter travel becomes
advantageous, for both predicted and empirical (ie historical) times.
Note that helicopter travel times are largely linear as expected, while
ground ambulance travel times generally have adownward slope as
distances increase - The general trend is likely due to ambualances
responding to more distant calls generally being able to travel faster.
The point as which these lines cross can generally be interpreted as the
“break-even” point where expected response- and transport times are
equal for ground and air ambulances (eg. incidents >25 km away from
an ambulance station may benefit in terms of response times from a
helicopter response).

```
timesvdistance <- select(pred_df,
                         id,
                         type,
                         StartPlace,
                         "Empirical_Time_To location" = true_time_loc,
                         "Empirical_Time_To hospital" = true_time_hosp,
                         "Predicted (LM)_Time_To location" = pred_time_loc,
                         "Predicted (LM)_Time_To hospital" = pred_time_hosp,
                         "Predicted (GB)_Time_To location" = predgb_time_loc,
                         "Predicted (GB)_Time_To hospital" = predgb_time_hosp,
                         "Predicted (LM)_Difference_To location" = pred_diff_loc,
                         "Predicted (LM)_Difference_To hospital" = pred_diff_hosp,
                         "Predicted (GB)_Difference_To location" = predgb_diff_loc,
                         "Predicted (GB)_Difference_To hospital" = predgb_diff_hosp,
                         "Empirical_Distance_To location" = drive_loc_distance,
                         "Empirical_Distance_To hospital" = drive_tot_distance,
                         "Predicted (LM)_Distance_To location" = drive_loc_distance,
                         "Predicted (LM)_Distance_To hospital" = drive_tot_distance,
                         "Predicted (GB)_Distance_To location" = drive_loc_distance,
                         "Predicted (GB)_Distance_To hospital" = drive_tot_distance) %>%
  st_drop_geometry() %>%
  pivot_longer(-c(type,id,StartPlace)) %>%
  separate(name,sep = "_",into = c("est","var","destination")) %>%
  pivot_wider(names_from = var,
              values_from = value,
              id_cols=c(type,destination,est,id,StartPlace))

timesvdistance %>%
  ggplot(aes(x=Distance,y=Time,color = type)) +
  geom_point(alpha = 0.1) +
  facet_grid(destination~est,scales = "free") +
  geom_smooth(data = mutate(timesvdistance,
                            Time = ifelse(Distance>60 & destination == "To location",NA,Time)))
```

```
## `geom_smooth()` using method = 'gam' and formula = 'y ~ s(x, bs = "cs")'
```

```
## Warning: Removed 4851 rows containing non-finite outside the scale range
## (`stat_smooth()`).
```

```
## Warning: Removed 4809 rows containing missing values or values outside the scale range
## (`geom_point()`).
```

```
timesvdistance %>%
  filter(!is.na(Difference)) %>%
  ggplot(aes(x=Distance,y=Difference)) +
  geom_point(alpha = 0.05) +
  facet_grid(destination~est,scales = "free") +
  geom_smooth(data = mutate(timesvdistance,
                            Difference = ifelse(Distance>60 & destination == "To location",NA,Difference)) %>%
                filter(!is.na(Difference)),
              method = "lm") +
  geom_hline(yintercept = 0, color = "red") +
  scale_x_continuous(breaks = seq(0,180,20)) +
  labs(x="Distance (km)",
       y= "Difference (minutes)")
```

```
## `geom_smooth()` using formula = 'y ~ x'
```

## Response time maps

To generate rasters of expected response times within the region,
thin plate splines were used to interpolate expected response times
based on the models reported above.

Note: Change eval = FALSE to TRUE when retraining models

```
generate_tps_df <- function(data,
                            time,
                            unit,
                            CloseDestination,
                            est,
                            grid = grid_inregion,
                            nmax=100){
  
  data <- data %>%
    group_by(latitude,longitude,geometry) %>%
    summarise(time = mean(eval(parse(text=time)),na.rm=T)) %>%
    filter(!is.na(time),
           !is.infinite(time)) %>%
    ungroup()
  
  fit <- Tps(st_coordinates(data), 
             data$time, 
             lambda = 0.001)
  
  grid_coords <- as.data.frame(st_coordinates(st_as_sf(grid)))
  
  preds <- predict(fit,grid_coords)
  
  tps_df <- as.data.frame(grid) %>%
    bind_cols(data.frame(time = preds))
  
  tps_df$unit <- unit
  tps_df$CloseDestination <- CloseDestination
  tps_df$est <- est
  
  return(tps_df)
}

# Create grid (dense matrix of lat/lon)
lon_range <- seq(min(combined_df$longitude)-0.1,
                 max(combined_df$longitude)+0.1,
                 length.out = 200)

lat_range <- seq(min(combined_df$latitude)-0.1,
                 max(combined_df$latitude)+0.1,
                 length.out = 300)

grid <- expand.grid(longitude = lon_range, latitude = lat_range)
coordinates(grid) <- ~longitude + latitude
gridded(grid) <- TRUE

grid_inregion <- expand.grid(longitude = lon_range,
                             latitude = lat_range)  %>%
  st_as_sf(x = .,
           coords = c("longitude", "latitude"),
           remove = F,
           crs = projcrs) %>%
  st_join(kommuner, join = st_within) %>%
  filter(!is.na(KnKod)) %>%
  mutate(point_id = row_number())

tps_df <- bind_rows(generate_tps_df(filter(pred_df,type=="amb"),
                                    time ="true_time_loc",
                                    unit="Ambulance",
                                    CloseDestination="To location",
                                    est = "Empirical"),
                    generate_tps_df(filter(pred_df,type=="amb"),
                                    time ="true_time_hosp",
                                    unit="Ambulance",
                                    CloseDestination="To hospital",
                                    est = "Empirical"),
                    generate_tps_df(filter(pred_df,type=="hkp",
                                           !is.na(true_time_hosp)),
                                    time ="true_time_loc",
                                    unit="Helicopter",
                                    CloseDestination="To location",
                                    est = "Empirical"),
                    generate_tps_df(filter(pred_df,type=="hkp",
                                           !is.na(true_time_hosp)),
                                    time ="true_time_hosp",
                                    unit="Helicopter",
                                    CloseDestination="To hospital",
                                    est = "Empirical"),
                    generate_tps_df(filter(pred_df,type=="amb"),
                                    time ="pred_drive_loc",
                                    unit="Ambulance",
                                    CloseDestination="To location",
                                    est = "Predicted (LM)"),
                    generate_tps_df(filter(pred_df,type=="amb",
                                           !is.na(true_time_hosp)),
                                    time ="pred_drive_hosp",
                                    unit="Ambulance",
                                    CloseDestination="To hospital",
                                    est = "Predicted (LM)"),
                    generate_tps_df(filter(pred_df,type=="hkp"),
                                    time ="pred_fly_loc",
                                    unit="Helicopter",
                                    CloseDestination="To location",
                                    est = "Predicted (LM)"),
                    generate_tps_df(filter(pred_df,type=="hkp",
                                           !is.na(true_time_hosp)),
                                    time ="pred_fly_hosp",
                                    unit="Helicopter",
                                    CloseDestination="To hospital",
                                    est = "Predicted (LM)"),
                    generate_tps_df(filter(pred_df,type=="amb"),
                                    time ="predgb_drive_loc",
                                    unit="Ambulance",
                                    CloseDestination="To location",
                                    est = "Predicted (GB)"),
                    generate_tps_df(filter(pred_df,type=="amb",
                                           !is.na(true_time_hosp)),
                                    time ="predgb_drive_hosp",
                                    unit="Ambulance",
                                    CloseDestination="To hospital",
                                    est = "Predicted (GB)"),
                    generate_tps_df(filter(pred_df,type=="hkp"),
                                    time ="predgb_fly_loc",
                                    unit="Helicopter",
                                    CloseDestination="To location",
                                    est = "Predicted (GB)"),
                    generate_tps_df(filter(pred_df,type=="hkp",
                                           !is.na(true_time_hosp)),
                                    time ="predgb_fly_hosp",
                                    unit="Helicopter",
                                    CloseDestination="To hospital",
                                    est = "Predicted (GB)"))


tps_df <- tps_df %>%
  st_as_sf() %>%
  mutate(
    longitude = st_coordinates(.)[, 1],
    latitude = st_coordinates(.)[, 2]
  ) 

save(tps_df,file = "tps_df.rda")
```

```
load("tps_df.rda")
```

The maps below represent average predicted response times- and
transport times within the region.

These times can then be differenced to generate a map identifying
areas where a helicopter response typically has the potiential to reduce
response and/or transport times to the hospital. In the below plot, both
Empirical and Predicted times are displayed as a form of validation.

```
tps_df_ambvhkp <- tps_df %>%
  pivot_wider(names_from = unit,
              values_from = time,
              id_cols = c(est,CloseDestination,geometry,latitude,longitude)) %>%
  mutate(Difference = Ambulance - Helicopter) %>%
  pivot_longer(-c(est,CloseDestination,geometry,latitude,longitude)) %>%
  mutate(time_cut = cut(value,c(-Inf,-5,5,10,20,30,Inf)))

tps_df_ambvhkp  %>%
  filter(name == "Difference") %>%
  ggplot(aes(x = longitude, 
             y = latitude, 
             fill = value)) +
  geom_raster(interpolate = F) + 
  scale_fill_gradientn(
    colours = rainbow(20),
    
  ) +
  facet_grid(est~CloseDestination) +
  labs(fill = "Minutes gained by\nhelicopter response") +
  geom_point(data = mutate(combined_df,
                           CloseDestination = ifelse(row_number()%%2==0,"To hospital","To location")),
             aes(x=StartLongitude,
                 y=StartLatitude),
             color="black",
             fill = NA) +
  geom_point(data = data.frame(StartLongitude = 17.601953300261158,
                               StartLatitude = 59.89143656746624,
                               CloseDestination = c("To hospital","To location")),
             aes(x=StartLongitude,
                 y=StartLatitude),
             color="blue",
             fill = NA) +
  theme_void()
```

If used in a practical application, such maps may be more easily read
if presented in a binned form:

```
diffmap <- tps_df_ambvhkp  %>%
  filter(name == "Difference",
         !is.na(time_cut)) %>% 
  ggplot(aes(x = longitude, 
             y = latitude, 
             fill = time_cut)) +
  geom_raster(interpolate = F) +
  scale_fill_manual(values=c("darkred","grey","lightblue","blue","darkblue","black")) +
  geom_point(data = mutate(combined_df,
                           CloseDestination = ifelse(row_number()%%2==0,"To hospital","To location")),
             aes(x=StartLongitude,
                 y=StartLatitude),
             shape = 21,
  fill = "white",
  colour = "black",
  size = 2,
  stroke = 1.5) +
  geom_point(data = data.frame(StartLongitude = 17.601953300261158,
                               StartLatitude = 59.89143656746624,
                               CloseDestination = c("To hospital","To location")),
             aes(x=StartLongitude,
                 y=StartLatitude),
  shape = 23,
  fill = "#FF00FF",
  colour = "black",
  size = 3,
  stroke = 1.5) +
  facet_grid(est~CloseDestination) +
  labs(fill = "Minutes gained by\nhelicopter response") +
  theme_void()

diffmap
```

It may be seen that in this region, there are few areas where a
helicopter response can be expected to arrive on scene quicker than an
ambulance, however, incidents in many outlying regions may benefit in
terms of reduced transport times to hospital. More accurate predictions
could be obtained by using a tool generating individual level
predictions when can take time of day, call characteristics, and current
ambulance availability into account.

# Variable importance metrics

```
# GEMS to location
xgb.importance(model = models$gb_drive_loc) %>% kable()
```

| Feature | Gain | Cover | Frequency |
| --- | --- | --- | --- |
| drive\_loc\_duration | 0.638 | 0.211 | 0.111 |
| drive\_loc\_distance | 0.257 | 0.144 | 0.083 |
| time\_theta | 0.021 | 0.121 | 0.128 |
| longitude | 0.018 | 0.073 | 0.101 |
| latitude | 0.014 | 0.063 | 0.112 |
| distance\_nearest | 0.007 | 0.058 | 0.072 |
| temperature | 0.007 | 0.043 | 0.057 |
| drive\_tot\_duration | 0.006 | 0.060 | 0.033 |
| distance\_hkp | 0.005 | 0.023 | 0.041 |
| snowdepth | 0.005 | 0.023 | 0.037 |
| drive\_hosp\_duration | 0.004 | 0.027 | 0.031 |
| distance\_hosp | 0.004 | 0.020 | 0.028 |
| drive\_hosp\_distance | 0.003 | 0.019 | 0.022 |
| visibility | 0.002 | 0.010 | 0.027 |
| windspeed | 0.002 | 0.008 | 0.021 |
| WeekDay | 0.001 | 0.010 | 0.020 |
| PrioOut | 0.001 | 0.032 | 0.017 |
| precipitation | 0.001 | 0.010 | 0.010 |
| distance\_tot | 0.001 | 0.004 | 0.011 |
| StartPlace | 0.001 | 0.011 | 0.012 |
| drive\_tot\_distance | 0.001 | 0.027 | 0.017 |
| CallType | 0.001 | 0.005 | 0.008 |

```
# HEMS to location
xgb.importance(model = models$gb_fly_loc) %>% kable()
```

| Feature | Gain | Cover | Frequency |
| --- | --- | --- | --- |
| time\_theta | 0.282 | 0.271 | 0.151 |
| distance\_hkp | 0.207 | 0.091 | 0.058 |
| distance\_tot | 0.153 | 0.027 | 0.010 |
| temperature | 0.044 | 0.091 | 0.083 |
| latitude | 0.041 | 0.069 | 0.144 |
| drive\_hosp\_distance | 0.039 | 0.018 | 0.023 |
| drive\_hosp\_duration | 0.029 | 0.015 | 0.030 |
| drive\_loc\_distance | 0.028 | 0.032 | 0.043 |
| distance\_nearest | 0.026 | 0.084 | 0.073 |
| drive\_loc\_duration | 0.022 | 0.088 | 0.086 |
| drive\_tot\_duration | 0.021 | 0.065 | 0.025 |
| windspeed | 0.021 | 0.019 | 0.048 |
| longitude | 0.021 | 0.033 | 0.065 |
| WeekDay | 0.017 | 0.020 | 0.048 |
| visibility | 0.013 | 0.031 | 0.030 |
| distance\_hosp | 0.012 | 0.014 | 0.013 |
| drive\_tot\_distance | 0.010 | 0.019 | 0.015 |
| PrioOut | 0.004 | 0.008 | 0.015 |
| StartPlace | 0.004 | 0.001 | 0.013 |
| CallType | 0.003 | 0.003 | 0.020 |
| snowdepth | 0.003 | 0.003 | 0.008 |

```
# GEMS to hospital
xgb.importance(model = models$gb_drive_hosp) %>% kable()
```

| Feature | Gain | Cover | Frequency |
| --- | --- | --- | --- |
| drive\_tot\_duration | 0.752 | 0.150 | 0.054 |
| drive\_tot\_distance | 0.066 | 0.053 | 0.031 |
| drive\_hosp\_duration | 0.029 | 0.108 | 0.063 |
| drive\_hosp\_distance | 0.029 | 0.051 | 0.034 |
| distance\_hkp | 0.014 | 0.059 | 0.059 |
| time\_theta | 0.013 | 0.078 | 0.109 |
| distance\_hosp | 0.013 | 0.028 | 0.038 |
| longitude | 0.010 | 0.068 | 0.077 |
| distance\_nearest | 0.010 | 0.073 | 0.067 |
| latitude | 0.009 | 0.045 | 0.089 |
| drive\_loc\_distance | 0.009 | 0.051 | 0.038 |
| temperature | 0.009 | 0.049 | 0.077 |
| drive\_loc\_duration | 0.009 | 0.043 | 0.054 |
| visibility | 0.007 | 0.016 | 0.050 |
| CallType | 0.005 | 0.023 | 0.021 |
| windspeed | 0.004 | 0.017 | 0.034 |
| snowdepth | 0.004 | 0.027 | 0.025 |
| distance\_tot | 0.004 | 0.034 | 0.027 |
| WeekDay | 0.002 | 0.004 | 0.025 |
| PrioOut | 0.002 | 0.015 | 0.018 |
| precipitation | 0.000 | 0.004 | 0.006 |
| StartPlace | 0.000 | 0.002 | 0.006 |

```
# HEMS to hospital
xgb.importance(model = models$gb_fly_hosp) %>% kable()
```

| Feature | Gain | Cover | Frequency |
| --- | --- | --- | --- |
| drive\_hosp\_distance | 0.294 | 0.058 | 0.031 |
| distance\_hosp | 0.141 | 0.046 | 0.022 |
| drive\_hosp\_duration | 0.120 | 0.081 | 0.044 |
| time\_theta | 0.111 | 0.241 | 0.160 |
| latitude | 0.051 | 0.071 | 0.142 |
| temperature | 0.049 | 0.147 | 0.084 |
| visibility | 0.038 | 0.049 | 0.053 |
| distance\_hkp | 0.037 | 0.059 | 0.067 |
| drive\_tot\_duration | 0.028 | 0.046 | 0.053 |
| longitude | 0.026 | 0.037 | 0.084 |
| WeekDay | 0.025 | 0.014 | 0.053 |
| drive\_loc\_duration | 0.021 | 0.045 | 0.044 |
| drive\_loc\_distance | 0.018 | 0.049 | 0.031 |
| distance\_nearest | 0.014 | 0.022 | 0.044 |
| drive\_tot\_distance | 0.010 | 0.005 | 0.009 |
| precipitation | 0.007 | 0.003 | 0.018 |
| windspeed | 0.004 | 0.016 | 0.022 |
| distance\_tot | 0.004 | 0.007 | 0.009 |
| snowdepth | 0.002 | 0.004 | 0.009 |
| CallType | 0.002 | 0.001 | 0.009 |
| StartPlace | 0.000 | 0.001 | 0.004 |
| PrioOut | 0.000 | 0.000 | 0.004 |
